# Global Research Trends, Hotspots, Impacts, and Emergence of Artificial Intelligence and Machine Learning in Health and Medicine: A 25-Year Bibliometric Analysis

**DOI:** 10.3390/healthcare13080892

**Published:** 2025-04-13

**Authors:** Alaa Dalky, Mahmoud Altawalbih, Farah Alshanik, Rawand A. Khasawneh, Rawan Tawalbeh, Arwa M. Al-Dekah, Ahmad Alrawashdeh, Tamara O. Quran, Mohammed ALBashtawy

**Affiliations:** 1 Department of Health Management and Policy, Faculty of Medicine, Jordan University of Science and Technology, Irbid 22110, Jordan; toquran19@med.just.edu.jo; 2 Department of Allied Medical Sciences, Faculty of Applied Medical Sciences, Jordan University of Science and Technology, Irbid 22110, Jordan; mhaltawalbih@just.edu.jo (M.A.); ratawalbeh@just.edu.jo (R.T.); aaalrawashdeh@just.edu.jo (A.A.); 3 Department of Computer Science, Faculty of Computer & Information Technology, Jordan University of Science and Technology, Irbid 22110, Jordan; fmalshanik@just.edu.jo; 4 Department of Clinical Pharmacy, Faculty of Pharmacy, Jordan University of Science and Technology, Irbid 22110, Jordan; rakhasawneh@just.edu.jo; 5 Department of Biotechnology and Genetic Engineering, Faculty of Science and Arts, Jordan University of Science and Technology, Irbid 22110, Jordan; amaldekah15@sci.just.edu.jo; 6 Department of Community and Mental Health Nursing, Princess Salma Faculty of Nursing, Al al-Bayt University, Mafraq 25113, Jordan; mohammadbash@aabu.edu.jo

**Keywords:** artificial intelligence, machine learning, medicine, health

## Abstract

**Background/Objectives**: The increasing application of artificial intelligence (AI) and machine learning (ML) in health and medicine has attracted a great deal of research interest in recent decades. This study aims to provide a global and historical picture of research concerning AI and ML in health and medicine. **Methods**: We used the Scopus database for searching and extracted articles published between 2000 and 2024. Then, we generated information about productivity, citations, collaboration, most impactful research topics, emerging research topics, and author keywords using Microsoft Excel 365 and VOSviewer software (version 1.6.20). **Results**: We retrieved a total of 22,113 research articles, with a notable surge in research activity in recent years. Core journals were *Scientific Reports* and *IEEE Access*, and core institutions included Harvard Medical School and the Ministry of Education of the People’s Republic of China, while core countries comprised the United States, China, India, the United Kingdom, and Saudi Arabia. Citation trends indicated substantial growth and recognition of AI’s and ML impact on health and medicine. Frequent author keywords identified key research hotspots, including specific diseases like Alzheimer’s disease, Parkinson’s diseases, COVID-19, and diabetes. The author keyword analysis identified “deep learning”, “convolutional neural network”, and “classification” as dominant research themes. **Conclusions**: AI’s transformative potential in AI and ML in health and medicine holds promise for improving global health outcomes.

## 1. Introduction

Over the course of several decades, artificial intelligence (AI) has developed to encompass increasingly sophisticated algorithms that function similarly to the human brain [1]. Similar to medical disciplines, artificial intelligence (AI) has numerous subfields, including computer vision, deep learning, and machine learning. The use of unique characteristics to find patterns that can be utilized to examine a given circumstance is known as machine learning. After that, the machine can “learn” from it and use that knowledge in similar situations in the future. Instead of using a static algorithm, this prediction tool can be actively used in clinical decision-making to customize patient care. Machine learning has developed into what is now called deep learning, which is made up of algorithms that build an artificial neural network (ANN) that can learn and make decisions on its own, much like the human brain [2,3,4]. A computer’s ability to learn and comprehend from a sequence of images or videos is known as computer vision [5].

The rapid advancement of artificial intelligence (AI) and machine learning (ML) is reshaping the landscape of healthcare, providing new opportunities for disease detection, diagnosis, and treatment optimization [6,7]. AI-driven algorithms are increasingly applied in medical imaging, predictive analytics, and personalized medicine [8,9,10], facilitating earlier and more accurate diagnosis, improved risk stratification, and timely intervention, advancing healthcare systems toward proactive disease management, reduced morbidity, and enhanced patient outcomes [11,12]. As AI continues to transform healthcare delivery, understanding the evolution of AI and ML research in medicine is crucial for identifying trends, emerging themes, and key contributors in this field.

As the scope of AI applications broadens, its impact on healthcare has garnered significant attention in the scholarly literature. With the rapid integration of AI into the medical domain, scholarly research on this subject has significantly expanded in recent years. This growing body of literature necessitates a comprehensive examination of research trajectories and emerging trends in AI and ML within health and medicine. Bibliometric analysis serves as a robust methodological approach for systematically evaluating global research trends, identifying emerging themes, and recognizing influential contributions across diverse disciplines [13,14,15,16]. Previous bibliometric studies have assessed AI or ML in health or medicine [17,18,19], or country-level contributions [20], but the global and comprehensive assessments of both AI and ML research trends in health and medicine remain limited. Accordingly, this study offers a comprehensive global analysis of both AI and ML research trends in the healthcare and medicine sector from 2000 to 2024. Specifically, this study aims to (1) evaluate annual publication and citation trends, (2) identify key contributors, including leading authors, countries, journals, and institutions, (3) assess highly cited publications and the evolution of thematic research over time, and (4) uncover contemporary research hotspots in AI and ML applications in health and medicine through an analysis of author keywords.

By providing a structured and data-driven overview of AI and ML research in healthcare, this study offers valuable insights for researchers, policymakers, and funding agencies. Understanding these research trends can inform future scientific endeavors and strategic investments, ensuring that AI-driven innovations continue to advance global health outcomes.

## 2. Materials and Methods

### 2.1. Study Design

This study is a comprehensive bibliometric, descriptive, and retrospective analysis of all original English-published articles that focused on AI and ML in health and medicine.

### 2.2. Database Used for Literature Retrieval

We retrieved and examined every research paper on AI and ML in health and medicine that was available in the Scopus database. One benefit of Scopus is that it includes PubMed and has twice as many indexed journals as Web of Science [21]. Because it retrieved the greatest number of publications, using the Scopus database alone is therefore justified. Numerous features in Scopus also make it easier to analyze citations, count research collaboration, and export data to Microsoft Excel for additional tabulation and mapping. Scopus has been utilized as the method to retrieve the necessary data in numerous published bibliometric research [14,15,16].

### 2.3. Search Strategy and Inclusion and Exclusion Criteria

This study employed a query in the search fields of the article title (Supplementary Material, Table S1). On 1 January 2025, a search strategy was carried out, created based on the search strategies of several previous bibliometric articles [18,22]. A question mark was used in the search strategy to capture a wide range of related keywords, and quotation marks were applied to retrieve exact phrases as specified in the search query. The final search strategy was employed after several preliminary attempts and validation processes. It included two main aspects: (AI and ML) and (health and medicine). Stringent inclusion and exclusion criteria were implemented to ensure both relevance and analytical focus. Inclusions involved articles published within the period from 1 January 2000 to 31 December 2024, to focus on contemporary research, articles written in English, and peer-reviewed original research articles. The exclusion criteria were duplicate articles, articles written in non-English languages, non-journal literature such as conferences, book series, trade journals, and undefined, and articles unrelated to AI/ML in health and medicine.

### 2.4. Validation of Data

To validate the data, title searches were performed to confirm the absence of false positives, with assistance from two colleagues in the medical fields. The retrieved articles were cross-referenced with highly cited publications in Google Scholar to enhance the comprehensiveness of the analysis. The consistency of the metadata and alignment with active journals and prolific authors further confirmed the credibility of the search approach.

### 2.5. Study Selection and Bias

Information such as the title, authors, institutions, document type, journal, DOI, abstract, publication year, organization, citations, keywords, open-access status, and funding details were extracted and saved as a CSV file for subsequent analysis. Articles were screened to exclude those out of scope, and any missing information was completed. Duplicate records were removed based on the title and DOI. To minimize potential bias, articles were ranked by citations, and the top 1000 most cited articles were reviewed to ensure relevance. We also compared the publication output of prolific authors with their contributions to the field to ensure accuracy and mitigate bias.

### 2.6. Scientific Literature Bibliometric Indicators

The cleaned list was exported to Microsoft Excel 395 and R package (v.4.2.2) “bibliometrix, Biblioshiny”. Advanced bibliometric indicators were calculated, including the total number of publications (TP), total citations (TC), average citations (AC), sole-authored publications (SA), co-authored publications (CA), number of contributing authors (NCA), annual collaboration index (ACI), number of cited publications (NCP), citations per cited publication (CCP), collaboration index (CI), collaboration coefficient (CC), number of active years of publication (NAY), productivity per active year of publication (PAY), average citation per year (AC/Y), publication year (PY-start), and author indices (h-index, g-index, and m-index). These indicators were measured and compared by the year of publication and citations. The 20 most prolific contributing countries, institutions, journals, authors, and publications were also identified and calculated with their relevant indicators.

### 2.7. Data Analysis and Mapping

Bibliometric analyses were conducted using Microsoft Excel 365, VOSviewer software (version 1.6.20), and the R package (v.4.2.2) “bibliometrix, biblioshiny”. After cleaning the data, we identified the most impactful and visible research topics and emerging research trends using Excel and RStudio. The “bibliometrix” format was employed to store the data, and the “biblioshiny” package was utilized to extract a range of features related to the research literature between [23,24].

We performed a comprehensive analysis and cartographic representation of the research landscape using VOSviewer [25], widely recognized for its capability to map and visualize the relationships between terms, authors, and research topics. It is particularly effective for visualizing co-authorship networks and co-occurrence data, which are essential for understanding the structure of research trends. By integrating Scopus data, we analyzed frequent author keywords and visualized research topics and their interrelationships. Nodes in the VOSviewer maps represent keywords, with their size reflecting frequency and their color indicating thematic clusters. Lines connecting nodes show relationships, with thicker lines indicating stronger associations. In addition, a co-authorship network visualization of authors and countries was conducted to identify international collaboration patterns.

Biblioshiny was utilized to visualize author productivity through Lotka’s law, highlighting the most productive authors. Further visualizations included the conceptual structural map, which encompassed both a thematic map and thematic evolution analysis.

The thematic map is a plot that was created on a two-dimensional matrix using the conceptual network. In this plot, the significance of each theme within the research area is based on its centrality, as demonstrated by the relevance of the keywords. Additionally, the development of each theme is indicated by its density, as measured by the degree of development [26,27]. Accordingly, themes were distributed into four demarcated quadrants, termed niche, basic, motor, and emerging/declining themes. The conditions for constructing the thematic map in this field included the author’s chosen keywords and a predetermined word count, which was set at 1000. Each bubble depicted in the plot represents a distinct network cluster, with the label assigned to the bubble corresponding to the word inside that cluster with the highest-frequency value. The relative size of each bubble corresponds to the frequency of the cluster words.

In addition to the thematic map, the thematic evolution showed the historical development of AI and ML in health and medicine-related publications. By analyzing the author keywords, the thematic evolution charts the progression of themes and how they have changed over time. This analysis was conducted using ’biblioshiny’ spanning over five distinct time segments. The division of time into these segments reflects the subjective judgment of the authors, aimed at better representing the evolution of themes.

## 3. Results

### 3.1. Bibliometric Analysis of All Articles Output

**Figure 1:**
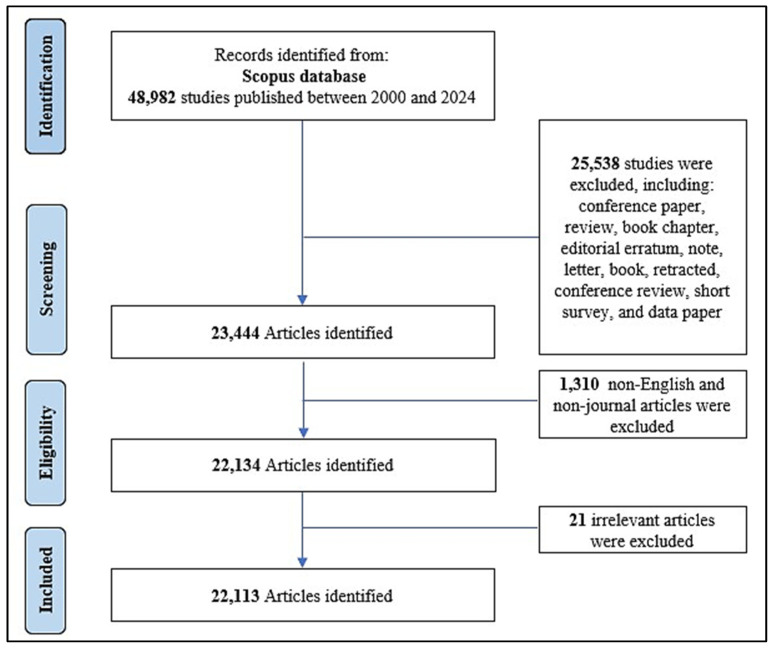
Flow chart of AI/ML in health and medicine-related literature search and screening.

The search strategy retrieved 22,134 articles, of which 21 were excluded by the manual screening (Figure 1). A total of 22,113 articles, spanning 25 active years, were included in the analysis. These articles collectively received 546,819 citations, with an average of 24.7 citations per article (Table 1). The research output demonstrates a steady annual growth rate of 24.8% and a high citation impact, as reflected in an h-index of 253 and a g-index of 410. The number of contributing authors was 76,167, indicating a higher level of collaboration and significant international collaboration, as evidenced by an international co-authorship rate of 264%.

### 3.2. Annual Publication and Citation Trends

**Figure 2:**
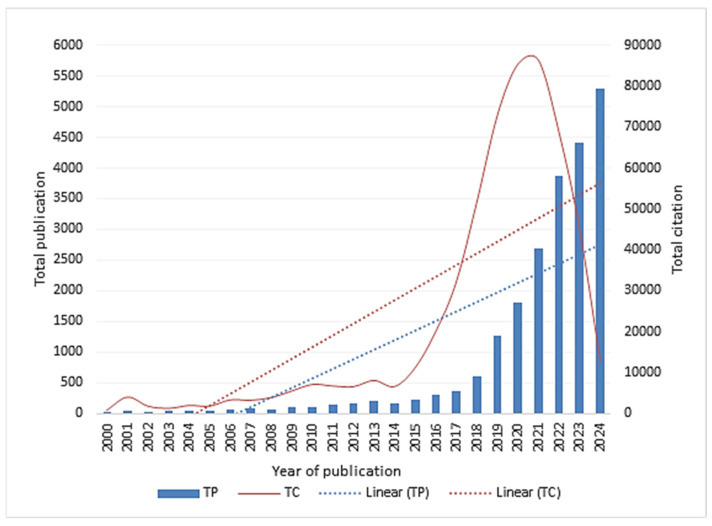
Annual publication and citation trends from 2000 to 2024.

Figure 2 presents the annual publication and citation trends from 2000 to 2024. The trend analysis indicates a steady increase in the total number of publications over time. The number of citations received by articles in this field has grown significantly over the years, starting from 694 in 2000 and reaching 86,284 in 2021. This indicates a substantial increase in the recognition and impact of the subject over time. The number of citations accelerated as the years progressed. For example, the number of citations increased dramatically from 2015. This linear growth suggests that the work gained increasing attention and influence as time went on. The steep increase in the number of citations, especially in the later years, resembles an exponential growth pattern. Exponential growth is an indication of widespread recognition of the topic investigated.

Table 2 presents the main bibliometric indicators of publications by the year of publication. Overall, all bibliometric indices have improved over the years of publication. The number of publications ranged between 26 in 2000 and 5300 in 2024.

### 3.3. Most Prolific Countries and Global Research Collaboration

As shown in Table 3, the United States emerges as the leading contributor to research in this field, accounting for 4752 articles, representing 21.5% of the total output. China ranks second with 4637 articles (21.0%), followed by India with 4000 articles (18.1%). The United Kingdom and Saudi Arabia also demonstrate notable contributions, with 1402 (6.3%) and 1128 (5.1%) articles, respectively.

**Figure 3:**
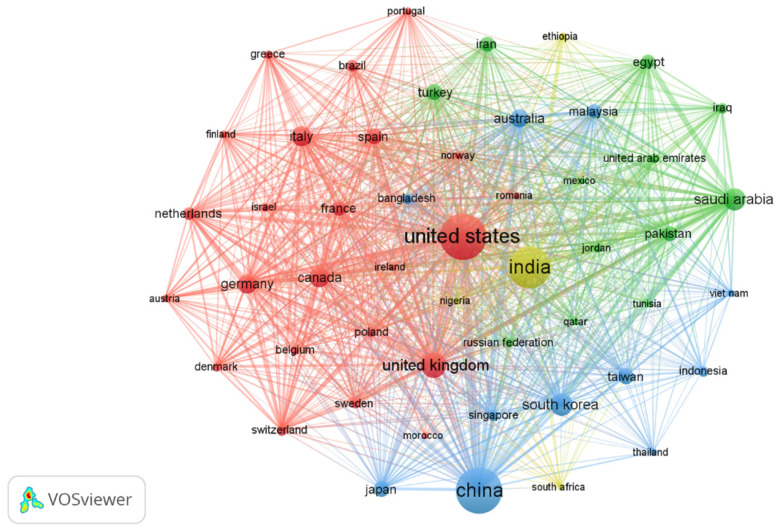
Network visualization map for co-authorship international collaboration. Minimum number of publications of a country = 88, minimum number of citations of a country = 0. Of the 139 countries, 50 meet the thresholds.

The network visualization map of countries, which considers countries with a minimum contribution of 88 articles, provides valuable insights into the dynamics of global research collaboration within a specific field (Figure 3). Within this map, a total of 50 countries are represented in this network, and noteworthy observations emerge. Primarily, the United States plays a significant role, exhibiting the highest number of connections and occupying a prominent position in the network. This highlights its significant research contributions and extensive collaborative efforts in the domain. The significant role of the United States in global research collaboration is evident from its extensive network connections (Links = 49, TLS = 3342), positioning it as a key facilitator of international scientific cooperation. A particularly noteworthy finding is the strong research partnership between the United States and India, as indicated by the substantial link strength between these nations (Links = 49, TLS = 1841). This reflects a high degree of scholarly engagement and exchange, reinforcing the global nature of scientific research. Additionally, the United Kingdom (Links = 49, TLS = 2125) and India (Links = 49, TLS = 1730) emerge as significant collaborators with the United States, further highlighting the importance of cross-border scientific cooperation in advancing research within this field. Interestingly, Saudi Arabia (Links = 49, TLS = 1706) stands out on the map with a large node size. This suggests that Saudi Arabia has made substantial contributions to research within the field and has its network of collaborations with various countries. Notably, the map highlights a particularly strong research partnership between Saudi Arabia and India. This collaboration underscores the active exchange of knowledge sharing and expertise between researchers and institutions in these two countries, further enriching the global research landscape in this domain. Cooperation between countries/regions has promoted the development of research.

### 3.4. Most Prolific and Impactful Institutions and Journals

According to the total number of publications, the top 20 institutions are listed in Table 4. With 292 papers (1.3%), Harvard Medical School was the most productive institution. The Ministry of Education of the People’s Republic of China came in second with 274 articles (1.2%), and the Chinese Academy of Sciences came in third with 219 articles (0.99%). Twenty institutions published at least 125 articles in all. It is interesting to note that six of the top 20 universities are in the US.

There were core journals that had a pivotal role in the field (Table 5). *Scientific Reports* led the pack with 521 articles, accounting for 2.4% of the publications in the field. Right on its heels is *IEEE Access*, contributing significantly with 491 articles, making up 2.2% of the articles. *PLoS ONE* takes a noteworthy position, featuring 289 articles (1.3%), while the *Applied Sciences (Switzerland)* journal follows closely with 203 articles (0.92%).

### 3.5. Authors’ Publication and Collaboration Analysis

Following Lotka’s law, we used Biblioshiny to analyze the distribution of author productivity and publication frequency to examine the relationship between authorship and research output. The findings indicate that most authors (n = 60,563) have contributed a single publication, while 14,816 authors have published between two and ten articles. In contrast, a small group of 788 authors has produced more than ten publications. Table 6 presents the 20 most prolific authors from a total of 76,167, ranked according to their total number of publications (TP). Each of these top authors has contributed at least 117 articles, with publication counts ranging from 117 to 347. Wang Y is identified as the most productive author (TP = 347; 2.0%), followed by Li Y (TP = 274; 1.8%) and Liu Y (TP = 256; 1.8%).

**Figure 4:**
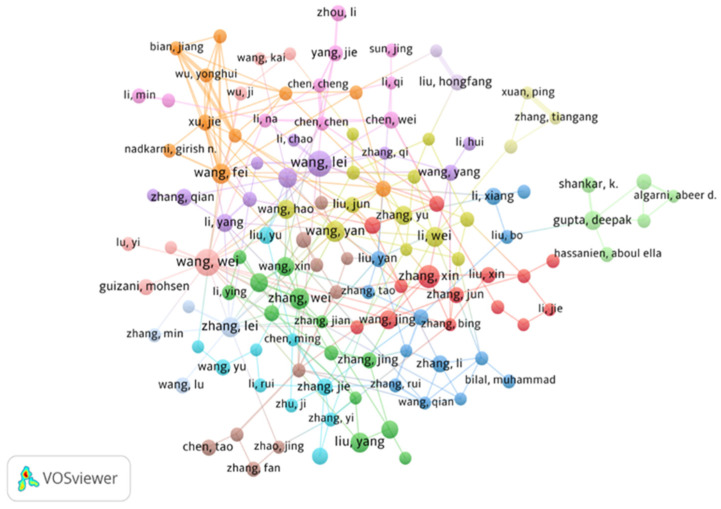
VOSviewer network of author co-authorship maps weighted by the number of articles (minimum number of publications of an author = 10, citation = 0, clusters = 14). Of the 76,167 authors, 174 met the thresholds: of them, 125 collaborated. Because some names may overlap, others may not be shown.

In terms of total citations (TC) and impact indices, which consider career length, Zhang Y emerges as the most influential author (TC = 9839; h-index = 38; g-index = 95), followed by Wang J (TC = 7994; h-index = 33; g-index = 86) and Liu Y (TC = 7853; h-index = 39; g-index = 84). To further refine the bibliometric assessment, a network visualization analysis was conducted (Figure 4). In the co-authorship network, authors are represented as nodes, where connections signify co-authored publications, and the thickness of the lines reflects the strength of collaboration. The analysis focuses on authors with a minimum of ten publications, without a citation threshold. Overall, the network map identifies 125 authors organized into 14 clusters, providing insights into collaborative structures within the field.

### 3.6. Most Impactful and Visible Research Topics

An article’s scientific impact and visibility are reflected in the number of citations it has received. The most influential research subjects were determined by analyzing the top 50 cited articles to determine which ones were the most visible and impactful in the area. Deep learning in medical imaging, disease prediction and diagnosis using AI, AI in electronic health record (EHR) analysis, AI for COVID-19 detection and diagnosis, explainability and trust in AI for healthcare, federated and privacy-preserving AI in medicine, personalized medicine and AI-based treatment optimization, and AI-powered genomics and drug discovery are the most significant and well-known research topics in AI and ML in medicine and health, according to the top 50 cited recent articles.

### 3.7. Emerging Research Topics

The dataset’s most recent publications featured several studies that integrate multiple emerging research areas. Among these studies and based on the recently published 50 articles, the most emerging research-related fields include AI and ML in disease prediction and diagnosis, explainable AI (XAI) in healthcare, AI in medical imaging and radiology, natural language processing (NLP) in smart healthcare, blockchain and cybersecurity in healthcare, IoT and edge AI in medical and bioinformatics applications, AI in precision medicine and biomarker discovery, AI for environmental and public health applications, AI in drug discovery and FDA-approved medical devices, AI in structural health monitoring, and smart cities.

### 3.8. Keyword Analysis

#### 3.8.1. Most Investigated Topics (Research Hotspots)

**Figure 5:**
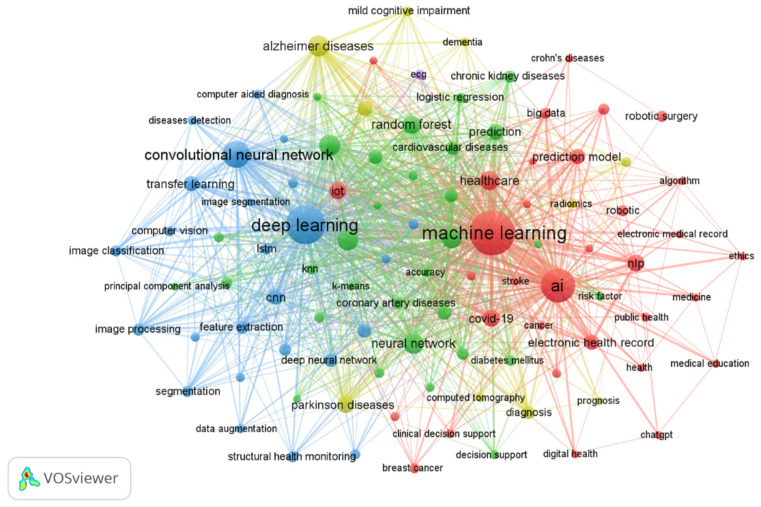
Co-occurrence analysis of authors’ keywords. The minimum number of occurrences of a keyword = 17; 100 meet the threshold.

By generating a network visualization map of author keywords, focusing on those appearing at least 17 times, we constructed a map comprising 100 distinct keywords (Figure 5). Within this network visualization map, the nodes rendered in the largest size correspond to the most commonly recurring items found within the literature, signifying key research focal points. The following list presents these prominent research hotspots, as visually depicted in the map, and based on the number of occurrences and TLSs provided by the VOSviewer program:

1. Machine learning and deep learning (8978 Occurrences, TLS: 13,113);
2. Artificial intelligence (2745 Occurrences, TLS: 3784);
3. Convolutional neural network (1540 Occurrences, TLS: 2393);
4. Classification (857 Occurrences, TLS: 1906);
5. Artificial neural network (775 Occurrences, TLS: 881);
6. Prediction and modeling (754 Occurrences, TLS: 1502);
7. Support vector machine (745 Occurrences, TLS: 1537);
8. Healthcare AND clinical decision support (706 Occurrences, TLS: 880);
9. Natural language processing (470 Occurrences, TLS: 672);
10. Electronic health record (384 Occurrences, TLS: 685);
11. Specific diseases (Ailments): Several specific diseases were explored in the dataset:
  - Alzheimer’s disease (823 Occurrences, TLS: 1552);
  - Parkinson’s disease (502 Occurrences, TLS: 804);
  - COVID-19 (479 Occurrences, TLS: 840);
  - Cardiovascular diseases (287 Occurrences, TLS: 603);
  - Heart diseases (275 Occurrences, TLS: 697);
  - Chronic kidney diseases (191 Occurrences, TLS: 360);
  - Coronary artery diseases (183 Occurrences, TLS: 324);
  - Breast cancer (125 Occurrences, TLS: 225);
  - Diabetes mellitus (123 Occurrences, TLS: 284);
  - Dementia (101 Occurrences, TLS: 245);
  - Depression (92 Occurrences, TLS: 165);
  - Crohn’s disease (79 Occurrences, TLS: 96);
  - Stroke (73 Occurrences, TLS: 94).

#### 3.8.2. Conceptual Structural Map of a Field: Thematic Map and Thematic Evolution

**Figure 6:**
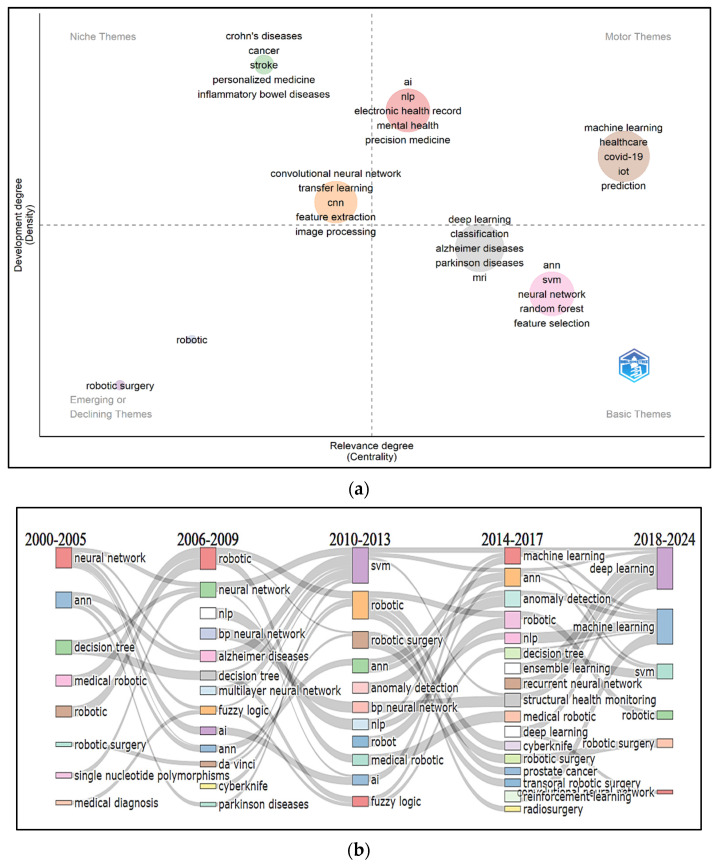
(**a**) Thematic analysis map developed by using a conceptual structure for authors’ keywords. (**b**) Thematic evolution of each topic and theme under five time slices. A longitudinal representation is adopted to facilitate an understanding of the tendencies of certain topics to merge with other themes or split into several other themes over the study period.

The primary goal of the thematic map examination was to intuitively analyze the development of themes in AI and ML-related research in health and medicine between 2000 and 2024. To do this, we built a two-dimensional matrix using the conceptual network and divided it into four distinct quadrants based on the centrality and density of themes (Figure 6a).

In the upper-left quadrant, labeled as ‘Niche Themes’, we find highly developed themes that remain isolated. These themes have strong internal connections but do not connect well with others, which limits their broader significance. The themes elucidated in this quadrant are related to several topics, the most frequent being “*Crohn’s disease*”, “*cancer*”, “*stroke*”, “*personalized medicine*”, “*inflammatory bowel diseases*”, and “*CNN*”. The lower-left quadrant, called ‘Emerging or Declining Themes’, highlights themes with both low density and centrality, suggesting they are not well-developed or may be losing relevance. This quadrant includes themes like “*robotic*” and “*robotic surgery*”. The upper-right quadrant, referred to as ‘Motor Themes,’ is where we see themes with both high density and centrality. These themes are well-developed, highly relevant, and connected to others. Key themes in this quadrant include topics like the “*electronic health record*”, “*mental health*”, “*precision medicine*”, “*COVID-19*”, and “*IoT*”. Finally, the lower-right quadrant, known as ‘Basic Themes,’ represents foundational and transversal themes. These are important for further study but have not been fully developed yet. The themes displayed in this quadrant are “*deep learning*”, “*classification*”, “*Alzheimer’s disease*”, “*Parkinson’s disease*”, “*MRI*”, “*SVM*”, “*neural networks*”, “*Random Forest*”, and “*feature selection*”.

Alluvial graphs were created by splitting the time into various time slices to better clarify the theme evolution within the subject field under investigation. We determined five time slices based on the distribution of articles annually, with four cutting points established in 2005, 2009, 2013, and 2017. Most of the extracted papers in the first time slice of 2000–2005 concentrate on the early applications of AI and robotics, as can be seen in Figure 6b: “*ANN*”, “*decision tree*”, “*robotic and robotic surgery*”, and “*medical diagnosis*”. In the second time slice (2006–2009), studies emphasize the expansion into medical AI and NLP. AI has begun to be applied in medical contexts, including neurodegenerative diseases (“*Alzheimer’s disease*” and “*Parkinson’s disease*”). Robotics, particularly in surgery (e.g., “*Da Vinci system*”), have gained prominence. In the third time slice (2010–2013), “*SVM*”, “*robotic surgery*”, “*anomaly detection*”, and “*fuzzy logic*” emerge as key themes. This period sees the rise of machine learning models and a focus on robotic-assisted surgery. In the fourth time slice (2014–2017), research continues to emphasize deep learning and AI in healthcare, with an increased focus on deep learning and neural networks in health applications, including cancer diagnosis, robotic surgery, and radiosurgery. In the last time slice (2018–2024), studies have a more oriented exploration of key themes: “*deep learning and machine learning*”, “*SVM*”, “*robotic surgery*”, and “*convolutional neural network*”.

## 4. Discussion

By examining the pertinent literature, we performed a bibliometric analysis for this study. From the standpoint of trends in the quantity of publications and citations, as well as the collaboration of nations and regions, the quantity of publications and collaboration of authors, journals, and cited journals, as well as keywords, we have examined the present research overall in this field. This study’s significance lies in its contribution to understanding the evolving trends, key contributors, and key research themes associated with the field. By employing bibliometric analysis, this study not only quantifies scholarly output but also identifies key research themes and their interrelationships. Furthermore, the incorporation of advanced metrics, such as keyword mapping, offers a multidimensional understanding of international research networking and research hotspots, contributing to the field’s advancement.

Regarding the number of publications, the yearly output of academic articles in this area generally showed an upward tendency, with a notable uptick in 2021 as a result of the COVID-19 pandemic. The fact that it continues to maintain a high number of publications suggests that this subject of study is still highly valuable and a hotspot. The upward trend in publications and citations aligns with the global increase in AI research in health and medicine [18].

The United States, China, and India were the top three countries in terms of publications and citations in the examination of nations in the research domain. This conclusion is consistent with a recent study that demonstrates the growing importance of donors from nations including the United States, India, and China [28]. This demonstrates the broad recognition of AI’s potential to significantly transform healthcare and underscores the importance of international collaboration in advancing the field. Furthermore, according to the most recent study, the United States has a major influence on the area and is now the top creator of scholarly papers [29]. The distribution of active countries serves as a stark reminder of the existing imbalance in research engagement between affluent destination nations and low- to middle-income source countries. Notably, scholarly contributions heavily favor WHO regions encompassing the Americas and Europe, while regions like Africa and the Eastern Mediterranean exhibit limited involvement in research endeavors. Several EMR countries, particularly low- and middle-income nations, are underrepresented in the research landscape. Factors such as political instability, limited funding, insufficient infrastructure, and lack of expertise hinder their participation. These challenges result in a low research output and limited visibility on the global stage. Efforts to enhance research capabilities in low-productive countries could include targeted funding, capacity-building programs, and the development of regional research networks [30]. Further, encouraging collaborations between well-resourced institutions and those in less developed areas can foster knowledge transfer and support local innovation [31,32]. The top countries must make a deliberate effort to share their knowledge and collaborate with these countries in future research endeavors.

The main groups in charge of advancing this area of study can be identified by looking at institutional connections. Outstanding research output is produced by establishments like King Saud University and Harvard Medical School. These important revelations support the development of interdisciplinary partnerships and cooperative networks to further research into AI-driven healthcare innovation. These findings are consistent with a prior study that indicated Harvard Medical School and King Saud University were the most productive institutions [28]. Furthermore, the Harvard Medical School had the most publications of any university, according to the most current study [29]. 

The thematic mapping revealed distinct research clusters, each representing a key thematic area in AI and ML research in healthcare. The map outlines the research themes and results in the identification of eight distinct thematic clusters that were micro-examined as follows:

The niche themes have two clusters with several hot topics as mentioned before in Figure 6a. The most cited studies within these clusters discuss the EMG-triggered robotic therapy system for stroke rehabilitation, which assists patient movements based on muscle activation to enhance motor recovery [33]. Another study presents a deep convolutional neural network for classifying interstitial lung disease patterns in CT images, achieving high accuracy in distinguishing between different lung abnormalities [34]. The topics in the motor themes are critical to the field and exhibit strong interconnections with other themes. These themes reflect ongoing trends in healthcare, where AI and ML play a significant role in improving diagnostic accuracy, treatment strategies, and patient outcomes. The most cited studies in those clusters explore AI applications in healthcare, categorizing them into virtual (informatics and decision support) and physical (robotics and nanotechnology) branches while addressing historical evolution, ethical concerns, and future implementation strategies [35]. A study presents a convolutional neural network-based multimodal disease risk prediction (CNN-MDRP) algorithm that analyzes both structured and unstructured hospital data to predict chronic diseases, achieving a prediction accuracy of 94.8% [36]. The emerging/declining themes have low centrality and low development, meaning they are either emerging or losing relevance. Despite their low density and centrality, these topics are still relevant in certain healthcare contexts. Future research could explore why these themes have not gained widespread traction and could involve investigating the technological, clinical, or financial barriers to their broader adoption. The most cited studies in those clusters explored and compared robotic versus fluoroscopy-guided pedicle screw insertion for metastatic spinal disease, assessing accuracy, safety, and clinical outcomes in a matched-cohort analysis [37]. They also evaluated the efficacy, benefits, and limitations of robotic and laparoscopic surgery for colorectal diseases, focusing on surgical precision, patient outcomes, and procedural feasibility [38]. The topics in the basic themes are fundamental to advancing AI and ML in healthcare and are crucial for future breakthroughs. Future research could focus on developing new deep learning architectures tailored to specific healthcare problems, such as improving early-stage diagnosis of diseases like Alzheimer’s or exploring novel classification methods for better medical imaging outcomes. The most impactful studies in those clusters explore adversarial attacks on deep learning-based medical image analysis systems, highlighting vulnerabilities, attack methods, and their implications for clinical reliability. It also discusses potential defense strategies to enhance the robustness and security of AI-driven medical imaging applications [39] and emphasizes the use of random forest-based similarity measures to improve the multi-modal classification of Alzheimer’s disease, enhancing diagnostic accuracy [40]. AI-driven models such as machine learning fuzzy logic are increasingly used for CKD detection, progression prediction, and personalized treatment plans [41,42]. AI-based radiology tools and predictive analytics are advancing the diagnosis and treatment of diseases such as tuberculosis, pneumonia, and influenza [43,44,45]. AI is being integrated into diagnostic processes for urinary tract infections (UTIs) and other related conditions, improving accuracy and early detection [46].

The thematic evolution shows a transition from basic neural networks and decision trees to advanced AI models, such as deep learning and reinforcement learning. Thematic shifts align with technological advancements, particularly in robotics and AI-driven healthcare solutions. The growing complexity of machine learning techniques indicates an increasing reliance on AI for precision medicine and automated decision-making. Recently, the combination of digitalization, artificial intelligence (AI), and robotics in healthcare has garnered considerable interest. These technological advancements are reshaping healthcare delivery, enhancing both care quality and efficiency. Recent research has emphasized the complex effects of these innovations. A recent study examines the relationship between AI, robotics, and policy within healthcare systems. It outlines how digitalization and AI can enhance care delivery and workforce interactions while providing policy suggestions for the smooth integration of these technologies into current healthcare structures. The study underscores the necessity of strategic alignment between technology and healthcare policy to achieve a fair and effective implementation across diverse healthcare sectors [47].

The current study looked closely at research trends and potentials. The use of AI in radiology is the subject of extensive research [48]. Present models concentrate on applying machine learning techniques to help process images, lower diagnostic mistakes, and increase the effectiveness of radiological imaging operations [49]. For instance, a study that was published in *Nature* showed that an AI model created by Google Health was able to identify breast cancer from mammograms more correctly than human radiologists [50]. The substantial potential of AI in the field of diagnostic imaging is demonstrated by this study [51]. Another significant area of study is pharmacogenomics, where AI offers individualized treatment plans according to a patient’s genetic composition, surroundings, and behavior. Advances in fields like pharmacogenomics are being used by AI-powered systems, like the AI tools that predict how patients will react to different cancer therapies, to create accurate predictive models of potential treatments [52]. A major trend during the COVID-19 pandemic was telehealth, where artificial intelligence (AI) improved remote patient monitoring (RPM), specifically virtual care [53]. The use of chatbots and virtual assistants in Babylon Health and other services is one example of this [54]. These technologies increase the population’s access to healthcare by giving consumers preliminary diagnoses and health recommendations. These subjects are seen as promising because they may help with some of the problems facing healthcare, like the increasing strain on healthcare systems, the need to raise service quality, and the need for individualized care. These themes, which highlight patient-centered, data-driven healthcare made possible by AI, have wide-ranging implications for future research. Developing these technologies, resolving their ethical issues, and incorporating them into other healthcare delivery systems will probably receive more attention. Growing interest in the topic and developments in other related areas of AI between 2015 and 2024 have led to a rise in articles about AI in healthcare.

It is clear from a closer look at the articles that a large amount of research and innovation has been concentrated on using machine learning (ML), especially in areas like medical imaging, diagnosis and treatment, and predictive analysis [55]. Large datasets, the processing power required to work with them, and improvements in algorithmic approaches have all contributed to the increased interest in these topics. The need to efficiently handle and glean insights from unstructured clinical data, like electronic health records (EHRs) and medical literature, has propelled the development of natural language processing [56].

Furthermore, the potential to enhance the AI-enabled functionality of robots has drawn attention to the field of healthcare robotics, including surgical assistant robots and other medical aid robots [57]. In healthcare systems, the introduction of new technologies like deep learning and data storage has simplified the process of resolving ever-more complex problems [58]. Furthermore, handling and analyzing enormous amounts of healthcare data has been made easier by the use of cloud computing and big data analytics. Research on innovative technological solutions for healthcare has expanded as a result of the COVID-19 pandemic’s increasing demand for digital health solutions. These elements have expanded the scope and perspectives of study in this quickly developing topic and raised the quantity of published resources pertaining to AI in healthcare.

### Strengths and Limitations

The use of advanced bibliometric tools like VOSviewer and the R package “bibliometrix” allowed for detailed mapping of research trends, collaboration networks, and thematic evolution. These methodologies provided a comprehensive view of the research landscape and facilitated the identification of emerging areas of interest. Regarding the study’s limitations, several factors deserve consideration. Firstly, the reliance solely on the Scopus database presents a limitation. This singular source, although extensive, may not encompass the entirety of relevant articles in the field, potentially introducing bias into the selection of materials. Additionally, language and publication bias could have influenced this study’s findings. The inclusion criteria restricting articles to the English language might have inadvertently excluded valuable research published in other languages, potentially introducing language-related biases. Moreover, focusing primarily on primary research articles might have omitted valuable insights from comprehensive review articles. Although efforts were invested in validating the data, there remains the possibility of missing relevant articles, and the validation process may not be entirely foolproof, introducing an element of uncertainty. The quality assessment of the selected studies introduces potential subjectivity. The “citation lag” phenomenon affects the citation counts of recent publications, as they have had less time to accumulate citations. This should be considered when interpreting citation-based metrics, especially for newer studies. Although the bibliometric methodology employed here has some limitations, we believe that these findings offer full use of information to scientists and funding bodies regarding publication trends and ongoing collaborative work in this research field.

The thematic evolution shows a transition from basic neural networks and decision trees to advanced AI models, such as deep learning and reinforcement learning. Thematic shifts align with technological advancements, particularly in robotics and AI-driven healthcare solutions. The growing complexity of machine learning techniques indicates an increasing reliance on AI for precision medicine and automated decision-making.

## 5. Conclusions

This study explores the role of AI in health and medicine, providing an overview of its current state, identifying emerging trends, and evaluating its progression over time.

Key research hotspots include AI applications in disease prediction, diagnosis, machine learning, deep learning, and specific conditions such as Alzheimer’s, Parkinson’s, COVID-19, and cancer. Future research should focus on optimizing AI algorithms, ensuring clinical validation, and addressing ethical and implementation challenges. Interdisciplinary collaboration among AI researchers, clinicians, and policymakers is essential for translating AI advancements into real-world healthcare solutions.

International collaboration is a significant aspect of AI research, with the United States playing a leading role alongside India, the United Kingdom, and China. This cooperation fosters knowledge exchange and enriches the research landscape. Moving forward, validating AI-driven models in clinical settings while addressing ethical considerations, patient privacy, and data security will be crucial. Educating healthcare professionals on AI-driven healthcare and building trust in AI systems are also vital for successful integration into clinical workflows.

Future research should prioritize transparency, interpretability, and ethical standards while ensuring AI models are trained on diverse datasets to mitigate biases. Further exploration of AI in predictive analytics, personalized medicine, and telehealth is necessary to address emerging technological concerns. Expanding bibliometric analyses beyond citation metrics would also enhance our understanding of AI research dynamics. By adhering to these guidelines, the academic community can contribute to the responsible and beneficial application of AI in healthcare.

## Figures and Tables

**Table 1:** Main bibliometric indicator for RCTs in DM research.

Indicator	Total
Productivity	
Number of total publications (all years)	22,113
Number of active years of publication (NAY)	25
Productivity per active year (PAY)	884.5
Annual growth rate %	24.8
Impact	
Total citations	546,819
Average citations per publication, %	24.7
Number of cited publications	18,817
Citations per cited publication	29.1
h-index	253
g-index	410
Authorship	
Co-authored publications	76,167
Sole-authored publications	962
Co-authors per publication	5.79
Collaboration	
Annual collaboration index	2.4
Collaboration index	5.9
Collaboration coefficient	0.8
International co-authorships %	26.4

**Table 2:** Bibliometrics by the year of publication for total publications.

Year	TP	TC	AC	SA	CA	NCA	ACI	NCP	CCP	CI	CC	h-Index	g-Index
2000	26	694	26.7	3	23	85	2.3	24	29.0	3.3	0.7	12	26
2001	47	3935	83.7	10	37	160	2.4	45	87.4	3.4	0.7	25	47
2002	33	1720	52.1	5	28	131	3.0	30	57.3	4.0	0.7	18	33
2003	34	1171	34.4	4	30	178	4.2	34	34.4	5.2	0.8	20	34
2004	38	1883	49.6	2	36	160	3.2	38	49.6	4.2	0.8	21	38
2005	43	1714	39.9	5	38	189	3.4	40	42.9	4.4	0.8	24	41
2006	66	3245	49.2	6	60	259	2.9	60	54.1	3.9	0.7	30	56
2007	74	3156	42.6	3	71	350	3.7	70	45.1	4.7	0.8	29	55
2008	64	3814	59.6	5	59	266	3.2	62	61.5	4.2	0.8	33	61
2009	99	5425	54.8	6	93	493	4.0	97	55.9	5.0	0.8	35	72
2010	107	7018	65.6	9	98	474	3.4	104	67.5	4.4	0.8	44	83
2011	139	6631	47.7	8	131	615	3.4	135	49.1	4.4	0.8	45	78
2012	159	6488	40.8	16	143	714	3.5	150	43.3	4.5	0.8	45	75
2013	196	7999	40.8	12	184	919	3.7	185	43.2	4.7	0.8	49	83
2014	172	6523	37.9	13	159	816	3.7	161	40.5	4.7	0.8	44	75
2015	229	11,085	48.4	17	212	1028	3.5	202	54.9	4.5	0.8	52	100
2016	297	19,979	67.3	10	287	1449	3.9	279	71.6	4.9	0.8	60	136
2017	370	32,073	86.7	16	354	1961	4.3	354	90.6	5.3	0.8	81	173
2018	603	51,591	85.6	31	572	3459	4.7	586	88.0	5.7	0.8	107	212
2019	1259	72,853	57.9	65	1194	7027	4.6	1185	61.5	5.6	0.8	134	222
2020	1800	85,379	47.4	69	1731	10,078	4.6	1741	49.0	5.6	0.8	136	210
2021	2689	86,284	32.1	87	2602	16,809	5.3	2634	32.8	6.3	0.8	120	182
2022	3860	68,541	17.8	163	3697	22,627	4.9	3680	18.6	5.9	0.8	85	123
2023	4409	44,917	10.2	172	4237	26,406	5.0	3974	11.3	6.0	0.8	69	96
2024	5300	12,701	2.4	225	5075	31,242	4.9	2948	4.3	5.9	0.8	29	40
Total	22,113	546,819	24.7	962	21,151	76,167	2.4	18,817	29.1	3.4	0.7	253	410

Notes: TP = Total number of publications, TC = Total citations, AC = Average citations, SA = Sole-authored publications, CA = Co-authored publications, NCA = Number of contributing authors, ACI = Annual collaboration index, NCP = Number of cited publications, CCP = Citations per cited publication, CI = Collaboration index, CC = Collaboration coefficient.

**Table 3:** A bibliometric analysis of the 20 most collaborated countries.

Productivity and Impact					International Collaboration		
Rank	Country	TP	TC	AC	Rank	Country	TLS
1st	United States	4752	167,308	35.21	1st	United States	3342
2nd	China	4637	118,208	25.49	2nd	United Kingdom	2125
3rd	India	4000	70,248	17.56	3rd	China	1841
4th	United Kingdom	1402	58,447	41.69	4th	India	1730
5th	Saudi Arabia	1128	25,171	22.31	5th	Saudi Arabia	1706
6th	South Korea	1054	29,684	28.16	6th	Germany	1167
7th	Canada	914	32,812	35.90	7th	Canada	1084
8th	Italy	869	25,438	29.27	8th	Italy	1065
9th	Germany	782	32,191	41.17	9th	Australia	946
10th	Australia	708	24,762	34.97	10th	Pakistan	931
11th	Turkey	607	17,932	29.54	11th	South Korea	787
12th	Taiwan	553	13,485	24.39	12th	Spain	744
13th	Japan	547	11,144	20.37	13th	Netherlands	691
14th	Spain	538	14,808	27.52	14th	Switzerland	670
15th	Pakistan	518	15,773	30.45	15th	France	640
16th	Iran	486	12,668	26.07	16th	Malaysia	605
17th	France	478	14,624	30.59	17th	Egypt	599
18th	Malaysia	440	10,748	24.43	17th	Taiwan	429
19th	Egypt	412	11,414	27.70	19th	Sweden	423
20th	Netherlands	385	12,095	31.42	20th	Singapore	409

Abbreviations: TP: Total Number of Publications, TC: Total Citations, AC: Average Citations, TLS: Total Link Strength.

**Table 4:** Bibliometric analysis of the 20 most prolific institutions.

Rank	Institution	TP (%)	Country
1st	Harvard Medical School	292 (1.32)	United States
2nd	Ministry of Education of the People’s Republic of China	274 (1.24)	China
3rd	Chinese Academy of Sciences	219 (0.99)	China
4th	King Saud University	205 (0.93)	Saudi Arabia
5th	University of Toronto	201 (0.91)	Canada
6th	K L Deemed to be University	191 (0.86)	India
7th	Vellore Institute of Technology	185 (0.84)	India
8th	Massachusetts General Hospital	172 (0.78)	United States
9th	Stanford University	168 (0.76)	United States
10th	SRM Institute of Science and Technology	152 (0.69)	India
11th	Princess Nourah Bint Abdulrahman University	151 (0.68)	Saudi Arabia
12th	Brigham and Women’s Hospital	141 (0.64)	United States
13th	University College London	139 (0.63)	United Kingdom
14th	University of Pennsylvania	132 (0.60)	United States
15th	University of California, San Francisco	131 (0.59)	United States
16th	Sichuan University	128 (0.58)	China
17th	Prince Sattam Bin Abdulaziz University	127 (0.57)	Saudi Arabia
18th	Chinese Academy of Medical Sciences & Peking Union Medical College	126 (0.57)	China
19th	King Abdulaziz University	125 (0.57)	Saudi Arabia
19th	Sun Yat-sen University	125 (0.57)	China

Abbreviations: TP: Total publications.

**Table 5:** Bibliometric analysis of the 20 most prolific journals.

SCR	Journal	TP	TC	AC	SA	CA	NCA	ACI	NCP	CCP	CI	CC	h -Index	g -Index
1st	*Scientific Reports*	521	13,257	25.4	3	518	4634	7.9	442	30.0	8.9	0.9	56	94
2nd	*IEEE Access*	491	21,536	43.9	15	476	2351	3.8	440	48.9	4.8	0.8	76	132
3rd	*PLOS ONE*	289	6591	22.8	1	288	1998	5.9	248	26.6	6.9	0.9	44	67
4th	*Multimedia Tools and Applications*	212	3798	17.9	12	200	702	2.3	199	19.1	3.3	0.7	34	54
5th	*Applied Sciences (Switzerland)*	203	3060	15.1	3	200	1022	4.0	175	17.5	5.0	0.8	29	46
6th	*Diagnostics*	188	2810	14.9	6	182	1146	5.1	166	16.9	6.1	0.8	27	42
7th	*Sensors*	168	4216	25.1	4	164	933	4.6	152	27.7	5.6	0.8	34	59
8th	*International Journal of Intelligent Systems and Applications in Engineering*	162	614	3.8	5	157	612	2.8	110	5.6	3.8	0.7	11	19
9th	*Computers in Biology and Medicine*	159	5059	31.8	4	155	905	4.7	145	34.9	5.7	0.8	39	65
10th	*International Journal of Advanced Computer Science and Applications*	158	1497	9.5	10	148	584	2.7	121	12.4	3.7	0.7	19	33
11th	*Expert Systems with Applications*	152	8117	53.4	6	146	627	3.1	148	54.8	4.1	0.8	49	86
12th	*Biomedical Signal Processing and Control*	149	3715	24.9	5	144	612	3.1	144	25.8	4.1	0.8	34	54
13th	*BMC Medical Informatics and Decision Making*	145	5757	39.7	1	144	1003	5.9	122	47.2	6.9	0.9	31	74
14th	*Journal of Medical Internet Research*	125	2756	22.0	2	123	943	6.5	116	23.8	7.5	0.9	25	48
15th	*Computational Intelligence and Neuroscience*	118	4203	35.6	12	106	595	4.0	116	36.2	5.0	0.8	27	63
16th	*JMIR Medical Informatics*	117	2078	17.8	1	116	1096	8.4	107	19.4	9.4	0.9	23	41
17th	*Neural Computing and Applications*	109	3779	34.7	7	102	420	2.9	102	37.0	3.9	0.7	32	59
18th	*Computer Methods and Programs in Biomedicine*	108	6701	62.0	1	107	607	4.6	101	66.3	5.6	0.8	41	81
18th	*Computers, Materials and Continua*	108	1550	14.4	3	105	619	4.7	96	16.1	5.7	0.8	23	34
20th	*Heliyon*	103	454	4.4	3	100	598	4.8	74	6.1	5.8	0.8	11	16

Abbreviations: SCR: Standard competition ranking. Equal countries were given the same ranking number, and then a gap was left in the ranking numbers; TP: Total number of publications; TC: Total citations; AC: Average citations; SA: Sole-authored publications; CA: Co-authored publications; NCA: Number of contributing authors; ACI: Annual collaboration index; NCP: Number of cited publications; CCP: Citations per cited publication; CI: Collaboration index; CC: Collaboration coefficient.

**Table 6:** Most relevant authors.

Rank	Author	TP	TC	AC	h-Index	g-Index	m-Index	PY-Start
1st	WANG Y	347	6907	0.05	42	72	2.1	2006
2nd	ZHANG Y	294	9839	0.03	38	95	2	2007
3rd	LI Y	274	6906	0.04	39	76	2.6	2011
4th	LIU Y	256	7853	0.03	39	84	3	2013
5th	LI J	233	4567	0.05	34	61	1.417	2002
6th	WANG J	221	4500	0.05	34	61	1.889	2008
7th	ZHANG X	219	4665	0.05	33	62	1.32	2001
8th	WANG X	217	7994	0.03	33	86	1.65	2006
9th	LI X	211	4037	0.05	30	58	1.579	2007
10th	ZHANG J	197	4217	0.05	34	59	2.125	2010
11th	CHEN Y	185	5212	0.04	29	69	1.813	2010
12th	WANG H	176	4051	0.04	33	59	2.063	2010
13th	WANG Z	169	3030	0.06	28	49	2.333	2014
14th	WANG L	166	5710	0.03	29	73	1.381	2005
15th	LIU X	158	6821	0.02	32	81	2.133	2011
16th	LIU J	152	4177	0.04	29	62	1.381	2005
17th	LI H	147	4812	0.03	37	67	1.947	2007
18th	CHEN X	143	3096	0.05	29	52	1.813	2010
19th	CHEN J	139	2597	0.05	27	45	2.25	2014
20th	LIU Z	117	2177	0.05	27	43	2.455	2015

Abbreviations: TP: Total number of publications; TC: Total citations; AC: Average citations; PY: Publication year.

## Data Availability

Data presented in this study are included in the article/Supplementary Materials; further inquiries can be directed to the corresponding author.

## Supplementary Materials

The following supporting information can be downloaded at https://www.mdpi.com/article/10.3390/healthcare13080892/s1, Table S1: Search results from Scopus database.

## Abbreviations

The following abbreviations are used in this manuscript:

AI	artificial intelligence
ML	machine learning
LD	linear dichroism
TP	total publications
TC	total citations
AC	average citations
SA	sole-authored publications
CA	co-authored publications
NCA	number of contributing authors
ACI	annual collaboration index
NCP	number of cited publications
CCP	citations per cited publication
CI	collaboration index
CC	collaboration coefficient
NAY	number of active years of publication
PAY	productivity per active year of publication
AC/Y	average citation per year
PY-start	publication year
EHR	electronic health record
NLP	natural language processing
*IoT*	Internet of Things
*CNN*	convolution neural network
*MRI*	magnetic resonance imaging
*SVM*	support vector machine
*ANN*	artificial neural network
