# Supplementary material for: Global Research Trends, Hotspots, Impacts, and Emergence of Artificial Intelligence and Machine Learning in Health and Medicine: A 25-Year Bibliometric Analysis
Source: Healthcare (Basel). 2025 Apr 13;13(8):892. doi: 10.3390/healthcare13080892 (PMC12026717; doi:10.3390/healthcare13080892)
Supplement: Supplementary file 1 [file healthcare-13-00892-s001.zip › healthcare-3535848-supplementary.pdf]

Global research trends, hotspots, impact, and emerging of artificial intelligence and machine learning in health and medicine: A 25-Year Bibliometric Analysis

Table S1: Search Results from Scopus database.

Number Search Query Result	Search query	Result
#1	TITLE ("Machine learning" OR "Deep learn*" OR "neural network*" OR "support vector machine" OR "random forest" OR "decision trees" OR "nearest neighbors" OR "k?means" OR "na#ve bayes" OR "Hierarchical clustering" OR "anomaly detection" OR "apriori algorithm" OR "reinforcement learning" OR "q? learning" OR "adversarial learning" OR "policy gradient" OR "policy optimi?ation" OR "natural language process*" OR "supervised W2 learning" OR "un#supervised W2 learning" OR "artificial intelligen*" OR perceptron OR "Dimensionality Reduction" OR "ensemble learning" OR "Gradient Boosting Machine" OR "Latent Dirichlet allocation" OR "Machine intelligen*" OR "artificial neural network*" OR "Robotic*" OR "thinking computer system" OR "fuzzy expert system*" OR "fuzzy logic*" OR "evolutionary computation" OR "intelligent system" OR "Machine intelligence" OR "Natural language process*" OR "hybrid intelligent system*")	**1,080,425**
#2	AND TITLE (disease* OR illness OR health-related OR medic* OR "medical diagnosis" OR treatment OR health* OR wellness OR "well-being")	**6,298,132**
#3	AND (LIMIT-TO (PUBYEAR , 2000) OR LIMIT-TO (PUBYEAR , 2001) OR LIMIT-TO (PUBYEAR , 2002) OR LIMIT-TO (PUBYEAR , 2003) OR LIMIT-TO (PUBYEAR , 2004) OR LIMIT-TO (PUBYEAR , 2005) OR LIMIT-TO (	

	PUBYEAR , 2006) OR LIMIT-TO (PUBYEAR , 2007) OR LIMIT-TO (PUBYEAR , 2008) OR LIMIT-TO (PUBYEAR , 2009) OR LIMIT-TO (PUBYEAR , 2010) OR LIMIT-TO (PUBYEAR , 2011) OR LIMIT-TO (PUBYEAR , 2012) OR LIMIT-TO (PUBYEAR , 2013) OR LIMIT-TO (PUBYEAR , 2014) OR LIMIT-TO (PUBYEAR , 2015) OR LIMIT-TO (PUBYEAR , 2016) OR LIMIT-TO (PUBYEAR , 2017) OR LIMIT-TO (PUBYEAR , 2018) OR LIMIT-TO (PUBYEAR , 2019) OR LIMIT-TO (PUBYEAR , 2020) OR LIMIT-TO (PUBYEAR , 2021) OR LIMIT-TO (PUBYEAR , 2022) OR LIMIT-TO (PUBYEAR , 2023) OR LIMIT-TO (PUBYEAR , 2024)) AND (LIMIT-TO (DOCTYPE , "ar")) AND (LIMIT-TO (SRCTYPE , "j")) AND (LIMIT-TO (LANGUAGE , "English")) AND (LIMIT-TO (PUBSTAGE , "final"))	
#4	#1+#2+#3	**22,150**
#5	After remove duplication	**22,113**
